# Genomic analysis of two all-stage stripe rust resistance genes in the Vavilov wheat landrace AGG40807WHEA1

**DOI:** 10.1007/s00122-025-04965-1

**Published:** 2025-07-09

**Authors:** Raghvendra Sharma, Chunhong Chen, Peng Zhang, Hemlata Bharti, Venu Kumaran Vikas, Michael Norman, Katherine Dibley, Adnan Riaz, Tim Hewitt, Sami Hoxha, Kerrie Forrest, Evans Lagudah, Harbans Bariana, Urmil Bansal, Lee Hickey, Sambasivam Periyannan

**Affiliations:** 1 Commonwealth Scientific and Industrial Research Organization Agriculture and Food, Canberra, ACT 2601 Australia; 2 Centre for Crop Science, Queensland Alliance for Agriculture and Food Innovation, The University of Queensland, Brisbane, QLD 4072 Australia; 3 Plant Breeding Institute, School of Life and Environmental Sciences, Faculty of Science, The University of Sydney, Cobbitty, NSW 2570 Australia; 4 Directorate of Medicinal and Aromatic Plants Research, Indian Council of Agricultural Research, Anand, GJ 387310 India; 5 Indian Agricultural Research Institute-Regional Station, Indian Council of Agricultural Research, Wellington, TN 643231 India; 6 AgriBio, Centre for AgriBioscience, Department of Energy, Environment and Climate Action, Bundoora, VIC 3083 Australia; 7 School of Agriculture and Environmental Science, The University of Southern Queensland, Toowoomba, QLD 4350 Australia; 8 Centre for Crop Health, The University of Southern Queensland, Toowoomba, QLD 4350 Australia; 9 Present Address: Centre for Protected Cultivation, Indian Agricultural Research Institute, Indian Council of Agricultural Research, New Delhi, 110012 India; 10 Present Address: Agriculture Victoria Research, Department of Energy, Environment and Climate Action, Bundoora, VIC 3083 Australia; 11 Present Address: Immunology and Infectious Diseases, John Curtin School of Medical Research, The Australian National University, Canberra, ACT 2601 Australia; 12 Present Address: School of Science, Hawkesbury Campus, Western Sydney University, Richmond, NSW 2753 Australia

## Abstract

**Key message:**

Comparative genomic analysis of two all-stage stripe rust resistance loci from Vavilov wheat landrace accession, AGG40807WHEA1, using Chinese Spring and 10 + hexaploid wheat genomes and validation of closely linked KASP markers.

**Abstract:**

The ongoing occurrence and spread of wheat stripe rust, caused by the fungal pathogen *Puccinia striiformis* f. sp. *tritici*, threatens the global food security. Cultivation of varieties with effective sources of resistance is often followed by the appearance of virulent pathotypes at various times after their introduction. This requires an ongoing search for new sources. Tests of 296 accessions from the Vavilov wheat landrace collection identified numerous lines with broadly effective all-stage stripe rust resistance. Genetic analysis of one of these accessions (Australian Grains Genebank number AGG40807WHEA1) identified two all-stage resistance genes, temporarily named *YrV1* and *YrV2*. The *YRV1* and *YRV2* loci were mapped to 3.48–3.98 and 730.2–731.2 Mb intervals in the short arm of chromosome 3B and the long arm of chromosome 7B, respectively. A comparative genomic analysis of the *YRV1* locus in the Chinese Spring and the 10 + wheat pangenome databases revealed genomic rearrangements and lack of sequences encoding a nucleotide-binding and leucine-rich repeat (NLR) domain protein. Sequences belonging to *NLR*-like genes were present in the *YRV2* region. Kompetitive allele-specific PCR (KASP) markers designed from SNPs *IWB71814* and *IWB69562*, located at 0.4 cM and 0.5 cM distal to *YrV1* and *YrV2*, respectively, were validated for marker-assisted selection using 123 hexaploid and 15 tetraploid wheat and 14 triticale cultivars. *YrV1* and *YrV2* genes are potentially valuable resources, and use of the closely linked molecular markers will expedite their deployment in breeding.

**Supplementary Information:**

The online version contains supplementary material available at 10.1007/s00122-025-04965-1.

## Introduction

Wheat (*Triticum aestivum*) is one of the most important food and feed crops worldwide, providing a major source of calories for humans and domestic animals. Globally, wheat production needs to be increased rapidly, as the human population is projected to reach 10 billion by 2050 (Hickey et al. 2019). That increase needs to be achieved despite ongoing constraints such as stripe rust caused by the biotrophic fungal pathogen, *Puccinia striiformis* f. sp. *tritici* (*Pst*). Frequent appearance and spread of new *Pst* pathotypes in leading wheat-producing regions pose a serious threat to global food security. Pathotypes that emerged after the beginning of the twenty-first century are adapted to warmer climatic conditions and generally have a wide range of virulence to currently deployed all-stage resistance (ASR) genes (Bouvet et al. 2022). These pathotypes spread rapidly and have caused epidemics in reputedly warmer continents such as Africa (Bouvet et al. 2022), South America and Australia (Ding et al. 2021). Although replacement of, or addition to, defeated genes with effective ASR genes remains common, the strategy needs to be strengthened by use of multiple effective resistance gene combinations and greater use of adult-plant resistance (APR). An additional concern is the recent reports of tolerance of some *Pst* isolates to frequently used fungicides (Cook et al. 2021; Zhan et al. 2022).

Among the potential sources of stripe rust resistance in breeding, the primary wheat gene pools of hexaploid and tetraploid wheat and their chromosomally homologous diploid relatives are preferred donors (Feuillet et al. 2008). Collections of landraces and varieties prior to the Green Revolution represent reservoirs of genetic variation in many traits, including disease resistance (Bansal et al. 2013; Marone et al. 2021). For example, tests of the wheat landrace collection assembled by A.E. Watkins during the first half of the 20th Century, identified stripe rust resistance genes such as *Yr47* (Qureshi et al. 2017), *Yr51* (Randhawa et al. 2014), *Yr57* (Randhawa et al. 2015), *Yr63* (Mackenzie et al. 2023), *Yr72* (Chhetri et al. 2023), *Yr80* (Nasbiyera et al. 2019) *Yr81* (Gessese et al. 2019) and *Yr82* (Pakeerathan et al. 2019). Putatively novel genes for stripe rust resistance were also detected in landrace collections maintained in China (Yao et al. 2021), Ethiopia (Yirga and Badebo 2021) and India (Kumar et al. 2016). The wheat germplasm collection assembled in Russia by N.I. Vavilov is another resource currently under investigation (Jambuthenne et al. 2022).

Along with the broader focus on identification of diverse sources of rust resistance in landraces, there has been rapid progress in the development of molecular markers linked with resistance genes to expedite their transfer to modern germplasm. Although linked markers have been used for indirect selection of resistance genes since the 1930s, the molecular methodologies evolved from isozymes to DNA blot assays (such as restriction fragment length polymorphisms) to the current single-nucleotide polymorphism (SNP)-based kompetitive allele-specific PCR (KASP) markers. Fluorescence-based PCR assays performed in KASP marker assays are rapid, cost-effective, and currently used extensively in wheat breeding (Kaur et al. 2020). Completion of the Chinese Spring (CS) hexaploid wheat reference genome assembly (International Wheat Genome Sequencing Consortium [IWGSC] 2018) enabled development of the 90K (Wang et al. 2014), 660K (Sun et al. 2020) and wheat barley 40K (Keeble-Gagnere et al. 2021) SNP arrays for rapid genotyping and selection of agronomic traits, including disease resistance.

Using the CS genome assembly as reference, the 10 + Wheat Genomes Project assembled the sequences of an additional 10 hexaploid wheat cultivars (Walkowiak et al. 2020). These latter resources permit rapid detection of haplotypes surrounding candidate genes in tetraploid and hexaploid wheat genomes (Walkowiak et al. 2020), whereas earlier studies depended on the sequenced genomes of wild wheat species (*T. urartu*, *Aegilops tauschii* and *T. dicoccoides*) and other Poaceae members such as barley (*Hordeum vulgare*), rice (*Oryza sativa*), maize (*Zea mays*), sorghum (*Sorghum bicolor*), and *Brachypodium distachyon* (Brenchley et al. 2012; Ling et al. 2013; Avni et al. 2017; Luo et al. 2017). For instance, the rust resistance genes *Yr9/Sr31/Lr26* (Mago et al. 2005)*, Yr18* (Bossolini et al. 2006)*, Yr26* (Wu et al. 2018)*, Yr36* (Fu et al. 2009)*, Sr2* (Kota et al. 2006)*, Sr35* (Zhang et al. 2010) and *Lr10* (Feuillet et al. 2003) were fine mapped using comparative genomic information from rice and *Brachypodium*. Currently, the near-complete genomic sequences of the tetraploid and hexaploid wheat and progenitor species, together with advances in DNA capture and sequencing techniques, enable the development of robust strategies for isolating rust resistance genes. Resistance gene enrichment and sequencing (RenSeq) enabled rapid analysis of sequences related to the nucleotide-binding and leucine-rich repeat (*NLR*) gene family, commonly involved in defense against diseases (Steuernagel et al. 2016; Arora et al. 2019). This method has enabled the identification of candidate genes for more than a dozen disease resistance loci in wheat (Zhang et al. 2020).

Here, we screened a panel of 296 accessions from the Vavilov wheat collection for resistance to stripe rust in Australia. Further, we used the 90K SNP array, RenSeq pipeline and CS and hexaploid wheat pangenome reference genomes to characterize two ASR genes from a Vavilov wheat landrace (WLA) accession (University of Queensland accession WLA153; Australian Grains Genebank [AGG] number AGG40807WHEA1), originally collected from India (Riaz et al. 2016).

## Materials and methods

### Plant materials

**Fig. 1:**
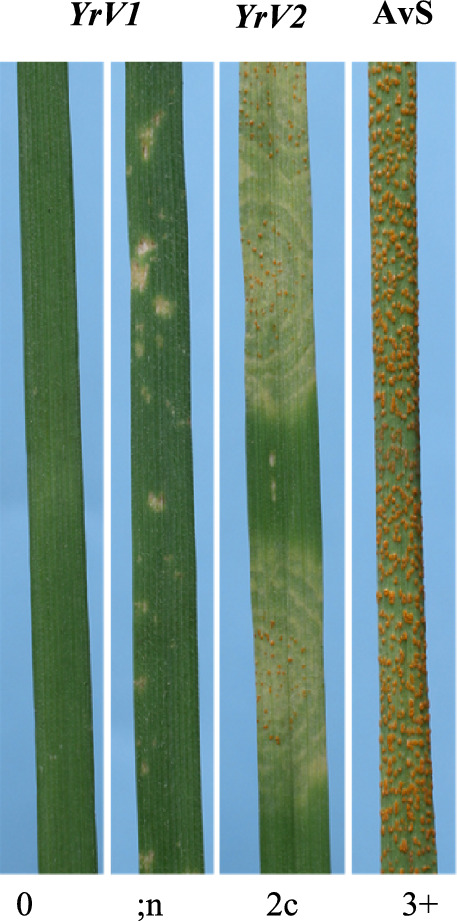
Infection types produced by seedlings with *YrV1* and *YrV2* when inoculated with *Pst* pathotype 150 E16 A + . Note the rippling effect surrounding the uredinia involving *YrV2*

The panel included 136 landraces, 32 cultivars, 10 breeding lines and 118 non-specified entries sourced through the Australian Grains Genebank. It included accessions from 28 countries across five continents, namely Africa, Asia, Europe, and the Americas (Riaz et al. 2016). Wheat lines carrying known ASR genes (*Yr1, Yr2, Yr3, Yr4, Yr5, Yr6, Yr7, Yr8, Yr9, Yr10, Yr15, Yr17, Yr25, Yr27, Yr32, Yr33, YrA, YrJ* and *YrT*) were used as differential controls and Avocet S (AvS) and/or Morocco were used as susceptible controls. Additional controls included Rubric (*Yr4*), *Yr57*/Hartog.17 (“/” denotes cross between the two lines and “.” denotes specific line from the cross), Sonora (*Yr58*), *Yr67*.43 and C591 (*Yr67*). One hundred and forty-seven F_3_ families derived from a cross of AGG40807WHEA1 and AvS were used in an initial genetic analysis of stripe rust response. Genomic DNA was extracted from 147 F_2_ plants using the method of Yu et al. (2017) and used to identify markers associated with stripe rust resistance. A second population of 131 lines was generated from an F_3_ family (AGG40807WHEA1/AvS.54) that segregated for a single gene conferring a distinctive low infection type (temporarily referred to as *YrV1*). Progeny from homozygous F_3_ family AGG40807WHEA1/AvS.75 with a similar low infection type served as the positive control for *YrV1*. Similarly, a population of 93 lines was generated from F_3_ family AGG40807WHEA1/AvS.57 that segregated for a distinctive intermediate infection type (temporarily referred to as *YrV2*) that showed a ‘rippling’ phenotype (Fig. 1). Homozygous F_3_ family AGG40807WHEA1/AvS.43 carrying only the second gene based on phenotype was selected as a positive control for multi-pathotype tests.

### Stripe rust pathotypes and phenotyping of stripe rust response

The wheat panel was initially screened with Plant Breeding Institute (PBI) *Pst* accession 617 of pathotype 134 E16 A + 17 + 27 + , a member of the post-2002 Australian lineage (Jambuthenne et al. 2022). Thirty-eight accessions showing resistance response to the *Pst* pathotype were selected for the current study and tested further with *Pst* accession 414, pathotype 104 E137 A + , a pre-2002 isolate. Panel members with a highly immune response against these pathotypes were tested for response to isolates of several additional pathotypes (Table 1). Virulence and avirulence profiles of all the *Pst* pathotypes used in this study can be found in Ding et al. (2021). Mapping populations were tested with pathotype 150 E16 A + (PBI accession 598), as it clearly distinguished between the two observed resistance phenotypes. The inoculation procedure followed Dracatos et al. (2016). Disease symptoms (infection types, ITs) were recorded about 14 days post-inoculation (dpi) using a 0–4 scale (McIntosh et al. 1995). The scale includes ITs from 0 to 4 with + or − signs and n (necrosis) and c (chlorosis) to indicate minor variations where IT 0 represents no visual evidence of infection,; for highly resistant flecking, 1 for resistant, 2 to 3 moderately resistant, and 3 + and 4 for susceptible and highly susceptible reactions, respectively. Responses on the first and second leaves were separated by “/”. Selected differential lines were included as controls in all tests.

**Table 1:** Infection types produced by six selected Vavilov wheat landrace (WLA) accessions when tested against 10 Australian *Pst* pathotypes (Plant Breeding Institute *Pst* accessions)

WLA No	AGG No	Pathotype and phenotype									
		Pre-2002				Post-2002					
		104 E137 A + (414)	104 E137 A- (515)	108 E141 A + (420)	110 E143 A + (444)	150 E16 A + (598)	134 E16 A + J + (602)	134 E16 A + J + 27 + (607)	134 E16 A + 17 + 27 + (617)	239 E237 A- 17 + 33 + (674)	198 E16 A + 17 + J + T + (687)
WLA028	AGG40691WHEA1	0	;1	0	0	0;	0; =	0	;n1 =	0;-	0;-
WLA101	Unknown	0	2-	;n	0;	;n	0	0	;n1 =	**3-**	**3**
WLA146	AGG40800WHEA1	0	**2 + **	;n	0	;n	;	0;	0;	**3**	**3**
WLA150	AGG40804WHEA1	0	1	0;	0	0;n	0,n	0	;n	**23-**	**2**
WLA151	AGG40805WHEA1	0	;1	;n	0;	;n	0;	0	0;	**23-**	**2**
WLA153	AGG40807WHEA1	0	;1	0;-	0	0	0	0	0;	**3–3**	**2 + **

Infection types that are different among the accessions are in bold

### Using molecular markers to postulate known *Yr* genes resistant to pre- and post-2002 *Pst* isolates

Wheat accessions resistant to both pre- and post-2002 *Pst* isolates were genotyped for molecular markers linked to known ASR genes that are resistant to both pre- and post-2002 *Pst* isolates such as *Yr1* (Bansal et al. 2009)*, Yr5* (Marchal et al. 2018)*, Yr15* (Klymiuk et al. 2018)*, Yr32* (Eriksen et al. 2004)*,* and *Yr33* (Zhou et al. 2022), and multi-pathogen adult-plant resistance (APR) genes *Yr18* (Krattinger et al. 2011)*, Yr29* (E. Lagudah, unpublished)*,* and *Yr46* (Moore et al. 2015) (Table S1). Polymerase chain reaction (PCR) assays were carried out in reaction mixtures containing 1 × GoTaq Flexi green buffer, 2.5 mM dNTPs, 200 nM of each forward and reverse primer, 1 U of Taq DNA polymerase (M829B; Promega, Madison, Wisconsin, USA), 50−100 ng genomic DNA, and brought to 20 µl volumes by adding autoclaved Milli-Q (Millipore Sigma, Burlington, MA, USA) water. PCR were performed in a Bio-Rad Laboratories (Hercules, CA, USA) thermal cycler using the following conditions: initial denaturation at 94 °C for 3 min, followed by 12 cycles of denaturation at 94 °C for 30 s, annealing starting at 67 °C (with a 1 °C decrease/cycle) for 30 s, and extension at 72 °C for 1 min. The program was repeated for 35 cycles at 94 °C for 30 s, 55 °C for 30 s, and 72 °C for 40 s; with a final extension of 72 °C for 5 min and incubation at 15 °C. PCR products were separated in 2% agarose gels. PCR-amplified fragment size was determined using a Gene Ruler 1 kb + DNA ladder (Thermo Fisher Scientific, Waltham, MA, USA) loaded with the samples.

### Bulked segregant analysis

Bulked segregant analysis (BSA, Michelmore et al. 1991) was carried out to identify the chromosomal regions of the targeted rust resistance gene(s). Genomic DNA bulks were prepared for each observed response category by pooling DNA from 10 homozygous lines with identical reactions. Total volumes of 100 µl DNA (~ 200 ng/µl) from each bulk was subjected to BSA using the Infinium 90K SNP chip (Wheat90k_ConsAkhunovKSU_15033654) platform developed by the AgriBio, Centre for AgriBioscience, Department of Energy, Environment and Climate Action, VIC 3083, Australia, to identify SNPs associated with resistance. Linkage analysis was performed computationally based on theta value, the probability that an allele from one sample was identical to an allele in another sample derived from the same population (Wang et al. 2014).

### Construction of linkage maps for the *YRV1* and *YRV2* loci

Marker sequences for the wheat 90K SNP chip were downloaded from the polymarker website (http://www.polymarker.info/) (Wang et al. 2014). The fluorescent FAM (GAAGGTGACCAAGTTCATGCT) and HEX (GAAGGTCGGAGTCAACGGATT) sequences of the tags were added upstream of allele-specific primers, as described previously (He et al. 2014). KASP assays were performed in 8 µl reaction mixtures containing 4 µl of 2 × KASP master mix, 0.11 µl KASP primer mix (12 µM of each forward and reverse primer, 30 µM of the common primer, and 46 µl Milli-Q water), 2 µl of ~ 25 ng/µl genomic DNA and 1.89 µl Milli-Q water. PCR was carried out with a Bio-Rad CFX96 real-time PCR system using the following protocol: 94 °C for 15 min, followed by 10 cycles of touchdown for denaturation at 94 °C for 20 s and 1 min for annealing at 65–57 °C (dropping 0.8 °C per cycle); followed by 32 cycles of 94 °C for 20 s (denaturation) and 57 °C for 1 min (annealing). Bio-Rad Laboratories CFX 3.1 Manager software was used to read the plate and analyze the allelic discrimination. KASP markers with clear polymorphisms between the resistant and susceptible parents were used to genotype the segregating populations (Tables S2 and S3). χ-squared tests were performed on data from the segregating populations to test the goodness-of-fit of observed and predicted segregation ratios. Linkage mapping was performed using MAP MANAGER v.QTXb20 (Manly et al. 2001). Genetic distances between molecular markers and resistance loci were calculated from recombination values using the Kosambi map function (Kosambi 1943). Declaration between markers and resistance loci was based on a default algorithm with a logarithm of odds (LOD) threshold of 3.0. Genetic linkage maps were drawn using MapChart software v.2.32 (Voorrips 2002).

### Generation of a mutant population for *YrV1*

A mutagenized population was generated by treating ~ 1,300 seeds of accession AGG40805WHEA1 (WLA151) with a 0.25% aqueous ethyl methanesulfonate (EMS) solution following Mago et al. (2017). AGG40805WHEA1 was used instead of AGG40807WHEA1 due to greater seed availability, a similar stripe rust response, and the presence of molecular markers flanking *YrV1* and *YrV2*. Further evidence arose from the allelism test, where there were hardly any susceptible lines identified in the testing of 200 F_2_ seeds from the cross between AGG40805WHEA1 and AGG40807WHEA1. A prior kill-curve analysis was performed to determine the EMS concentration causing a 50% reduction in plant survival. M_1_ plants were grown in the field, and a single spike was harvested from each plant to generate M_2_ families. Approximately 700 M_2_ lines (15 seedlings per line) were tested for response to pathotype 150 E16 A + . Stripe rust susceptible (mutant) individuals from each segregating family were progeny tested to confirm the mutation events and establish homozygous susceptible M_3_ lines.

### Candidate gene prediction for *YrV1* by mutagenesis, resistance gene enrichment and sequencing (MutRenSeq)

Genomic DNA from leaves of one-month-old susceptible mutant M_2_ plants was extracted and assayed using markers flanking the *YRV1* and *YRV2* locus to identify deletions. MutRenSeq analysis was conducted as described by Steuernagel et al. (2016). To capture *NLRs* from resistant wild type and mutant plants, a bait library was prepared at Arbor Biosciences (Ann Arbor, MI, USA) using a NEBNext Ultra Library Prep Kit (New England Biolabs, Ipswich, MA, USA). Targeted enrichment was carried out using the Mybaits protocol (MYcroarray). The *NLR*-enriched libraries were sequenced on a myReads NovaSeq SP500 platform to generate paired-end (PE 250) reads. Candidate *NLR* genes for the targeted resistance locus were predicted by analyzing the reads using the mutant hunter pipeline (https://github.com/TC-Hewitt/MuTrigo) (Hewitt et al. 2021). A de novo assembly was generated for reads captured from the wild-type sample using the CLC Assembly Cell (http://www.clcbio.com/products/clc-assembly-cell/) with default parameters. Trimmed reads from the wild-type and mutant lines were then aligned to the wild-type assembly to identify *NLR* contigs with deletions or a SNP difference between the wild-type and mutant lines.

### Comparative genome analysis of the *YrV1* and *YrV2* regions in the CS reference genome and 10 + wheat genomes project

To identify the physical positions of the stripe rust resistance gene loci, marker sequences flanking *YRV1* and *YRV2* were BLASTed against the CS v.2.0 reference genome sequence (https://urgi.versailles.inra.fr/blast/?dbgroup=wheat_iwgsc_refseq_v1_chromosomes&program=blastn) and 10 + Wheat Genomes Project database (https://www.wheatinitiative.org/). High-confidence gene sequences were extracted from the intervals between the two closest flanking markers for each gene. Disease resistance gene-like sequences were shortlisted to predict *YrV1* and *YrV2* candidates using an NCBI BLAST search.

### Validation of KASP markers closely linked to the *YrV1 *and *YrV2* resistance genes

Markers closely flanking *YRV1* and *YRV2* were validated using 123 hexaploid and 15 tetraploid wheats accessions, as well as 14 triticale cultivars (Table 2) (Norman et al. 2024). Rust resistance gene(s) and resistance responses of these lines were detailed in Park et al (2019). The KASP marker linked with *YrV2* was also tested on a *T. aestivum* ssp *sphaerococcum* panel consisting of 72 accessions (Table S4), as *Yr67*, a named ASR stripe rust resistance gene with a similar resistance response, was mapped to the same locus as *YRV2* and identified from an old wheat cultivar in India, where the dwarf wheat *T. aestivum* ssp *sphaerococcum* is prevalent. Accession AGG40807WHEA1 was used as the positive control for *YrV1* and *YrV2,* whereas AvS served as the negative control.

**Table 2:** Validation of the KASP markers *IWB71814* and *IWB69562* linked with *YrV1* and *YrV2*, respectively, on Australian tetraploid and hexaploid wheat and triticale cultivars

Cultivar type	Details	*IWB71814* allele	*IWB69562* allele
Hexaploid wheat	AGG40807WHEA1 (positive control for *YrV1* and *YrV2*) DS Bennett, Forrest, RGT Zanzibar, Shield and SQP Revenue	T:T	T:T
	Kiora, LG Gold, RGT Calabro and Viking	T:T	G:G
	Catapult, Condo, Correll, Cutlass, DS Pascal, EGA Wedgetail, Espada, Grenade CL Plus, Harper, Illabo, Impress CL Plus, Justica CL Plus, Kinsei, Kord CL Plus, LRPB Gazelle, LRPB Impala, LRPB Lancer, LRPB Oryx, LRPB Reliant, LRPB Scout, LRPB Trojan, Manning, Naparoo, Orion, Razor CL Plus, Steel, Sunguard, Yitpi and Zircon	C:C	T:T
	Avocet S, Morocco, Anapurna, Axe, B53, Beckom, Borlaug 100, Bremer, Buchanan, Calingiri, Chara, Chief CL Plus, Coolah, Corack, Cosmick, Derrimut, Devil, DS Darwin, DS Faraday, DS Tull, EG Jet, EG Titanium, EGA Bounty, EGA Eagle Rock, EGA Gregory, EGA Kidman, Einstein, Elmore CL Plus, Emu Rock, Estoc, Hartog, Hatchet CL Plus, Hydra, Jade, Janz, Livingston, Longsword, LRPB Arrow, LRPB Beaufort, LRPB Cobra, LRPB Dart, LRPB Flanker, LRPB Gauntlet, LRPB Havoc, LRPB Kittyhawk, LRPB Mustang, LRPB Nighthawk, LRPB Nyala, LRPB Parakeet, LRPB Spitfire, Mace, Magenta, Merlin, Mitch, Ninja, Phantom, Preston, RGT Accroc, RGT Ivory, RockStar, Scepter, SEA Condamine, SF Adagio, SF Ovalo, SF Scenario, Shark, Sheriff CL Plus, Strzelecki, Sunchaser, Sunlamb, Sunmate, Sunmax, Sunprime, Suntime, Suntop, Sunvale, Supreme, Tenfour, Tungsten, Tungsten, Vixen, Wallup, Westonia, Wyalkatchem and Zen	C:C	G:G
Durum wheat	Bitalli	T:T	G:G
	Caparoi, DBA Artemis, DBA Aurora, DBA Bindaroi, DBA Spes, DBA Vittaroi, Hyperno, Rotini and Tijlkuri	C:C	T:T
	DBA Lillaroi, EGA Bellaroi, Jandaroi and Penne	C:C	G:G
Triticale	Bison	T:T	G:G
	Astute, Berkshire, Canobolas, Cartwheel, Chopper, Endeavour, Fusion, Goanna, Joey, Kokada, Normandy, Wonambi and Yowie	C:C	G:G

## Results

### Vavilov wheat landrace accessions resistant to multiple *Pst* pathotypes

Among the 38 accessions resistant to *Pst* pathotype 134 E16 A + 17 + 27 + accessions AGG40691WHEA1 (WLA028), WLA101 (AGG number unknown), AGG40800WHEA1 (WLA146), AGG40804WHEA1 (WLA150), AGG40805WHEA1 (WLA151), and AGG40807WHEA1 (WLA153) displayed low infection types (ITs), 0 to 1 + , when infected with pathotype 104 E137 A + . Accessions AGG40720WHEA1 (WLA057) and AGG40772WHEA1 (WLA115) developed intermediate response (IT 2 +), whereas the remaining 13 and 17 accessions developed ITs 3 and 3 + , respectively, and were considered susceptible (Table S5). The first six accessions also displayed very low responses (IT 0 to;n) to five additional pathotypes (Table 1, Fig. S1). Only AGG40691WHEA1 developed a strong immune response to pathotypes 239 E237 A- 17 + 33 + and 198 E16 A + 17 + J + T +), whereas the other five accessions developed intermediate responses (IT 2 to 3) (Table 1). Four (AGG40800WHEA1, AGG40804WHEA1, AGG40805WHEA1 and AGG40807WHEA1) of the six accessions were originally collected in India, AGG40691WHEA1 was from Pakistan (earlier, India) and WLA101, a tetraploid line, had an unknown origin (Riaz et al. 2016).

### Genotyping of six resistant accessions with DNA markers linked to known *Yr *genes resistant to pre- and post-2002 *Pst* isolates

All six highly resistant accessions failed to amplify markers linked to known ASR genes *Yr1, Yr5, Yr15, Yr32* and *Yr33*, which are resistant to both pre- and post-2002 *Pst* isolates, raising the possibility that these accessions carried a new resistance gene(s). Marker analyses predicted *Yr18* in all six accessions, *Yr29* in durum accession AGG40691WHEA1 and *Yr46* in AGG40800WHEA1, AGG40804WHEA1, AGG40805WHEA1 and AGG40807WHEA1. The presence of one or other of these genes can have modifying effects on the responses of ASR genes.

### Genetic analysis indicates two ASR genes in AGG40807WHEA1

In the propagation of 200 F_2_ seeds from AGG40807WHEA1/AvS cross, we were able to harvest heads only from 147, as the remaining ~ 50 lines were severely necrotic and failed to survive after the seedling stage possibly due to the presence of progressive necrosis gene *Ne1* in the resistant parent in a heterogenous manner. The 147 AGG40807WHEA1/AvS F_2:3_ families inoculated with pathotype 150 E16 A + segregated digenically. Genotyping of the F_2_ population based on the F_3_ family (~ 30 plants per family) responses indicated segregation at two independent loci (χ^2^_4(34):2(15):4(44):2(17):1(7):2(21):1(9)_ = 0.775: P_6df_ = 0.775; Table S6). One of these genes conferred IT 0 to ;n, was temporarily named as *YrV1*, while the other conferred IT 2c with a distinctive rippling pattern was named *YrV2.* Lines lacking both genes were susceptible with IT 3 + and was similar to the susceptible parent AvS (Fig. 1).

### Mapping of *YrV1*

The 131 lines derived from F_3_ family AGG40807WHEA1/AvS.54 (IT 0;:3 +) showed segregation for *YrV1* [29 homozygous resistant (IT 0;), 60 segregating (IT 0; and 3 +) and 42 homozygous susceptible (IT 3 +); χ^2^_1:2:1_ = 3.5, P_1df_ ≥ 0.05]. BSA with the Infinium 90K SNP chip predicted 18 and 43 SNPs showing strong and moderate linkage, respectively, with *YRV1*. Among the 18 strongly linked SNPs, eight, six and four were located on chromosome arms 3BS, 7BS and 7AL, respectively. For the short arm of chromosome 3B, in addition to the high number of strongly linked SNPs, another six SNPs with moderate association and one with weak linkage to *YRV1* were also identified. KASP primers were designed for the 14 chromosome 3B-specific SNPs and four markers showed polymorphism between the parents AGG40807WHEA1 and AvS. Using the CS v.1.0 genome assembly, 40 additional SNP markers were selected randomly from the chromosome 3BS region to identify markers closely linked with *YrV1*. Finally, eight KASP markers (Table S2) were used to genotype the AGG40807WHEA1/AvS F_2_ population.

**Fig. 2:**
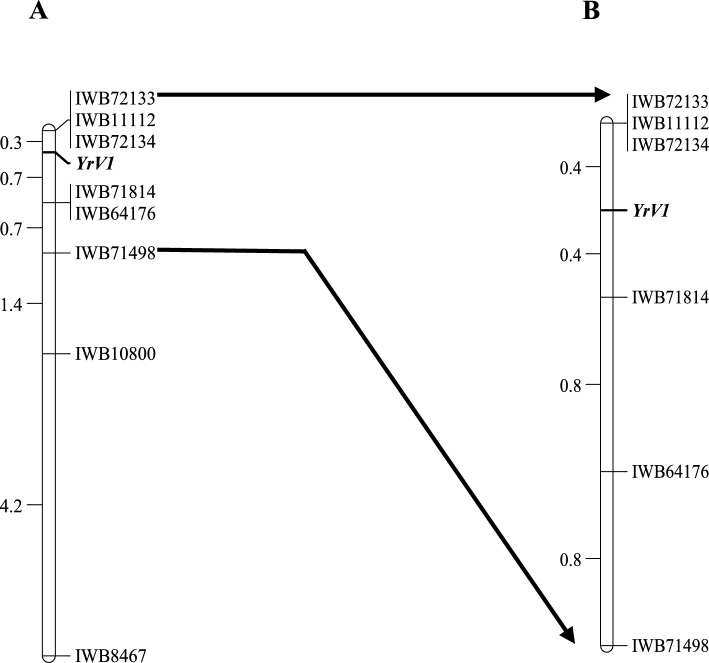
Linkage maps for the *YRV1* locus on chromosome arm 3BS. The maps were generated from **A** AGG40807WHEA1/AvS F_2:3_ and **B** AGG40807WHEA1/AvS.54 population

The eight KASP markers mapped to a 7.3 cM region, with *IWB72133*, *IWB11112* and *IWB72134* clustered 0.3 cM distal to the *YRV1* locus. Markers *IWB71814* and *IWB64176* were 0.7 cM proximal to *YRV1.* Marker, *IWB71498* mapped 0.7 cM away from markers *IWB71814* and *IWB64176* (Fig. 2). These six KASP markers were also genotyped on the 131 lines derived from AGG40807WHEA1/AvS.54. Markers *IWB72133*, *IWB11112* and *IWB72134* remained clustered at 0.4 cM distal to *YRV1,* whereas the proximal markers *IWB71814* and *IWB64176* were 0.4 cM and 1.2 cM away from *YrV1* (Fig. 2B).

### Relationship of *YrV1* with *Yr4*, *Yr57* and *Yr58* also located in chromosome 3B

As resistance genes *Yr4* (Bansal et al. 2010)*, Yr57* (Randhawa et al. 2015), and *Yr58* (Chhetri et al. 2016) were previously mapped to chromosome 3B, a comparison of responses of lines bearing these genes was made between AGG40807WHEA1/AvS.75 (carrying *YrV1*) and wheat lines Rubric (*Yr4*), *Yr57*/Hartog.17 (*Yr57*), and Sonora (*Yr58*) using four *Pst* pathotypes (Table 3). Rubric (*Yr4*) and Sonora (*Yr58*) showed resistance only to 134 E16 A + 17 + 27 + and were clearly different from AGG40807WHEA1/AvS.75. Line *Yr57*/Hartog.17 (*Yr57*) showed a susceptible response to 239 E237 A- 17 + 33 + , but high levels of resistance against the other three pathotypes. AGG40807WHEA1/AvS.75 (*YrV1*) was highly resistant (IT 1c) to 134 E16 A + 17 + 27 + , but displayed moderate resistance to the remaining pathotypes (Table 3, Fig. S2). The difference in response could be due to variation in the genetic backgrounds as *Yr57*/Hartog.17 (*Yr57*) was positive for markers *IWB72134* and *IWB71814* flanking *YrV1,* indicating that *Yr57* and *YrV1* might be the same gene.

**Table 3:** Multi-pathotype analysis of *YrV1* and *YrV2* with *Yr* genes mapped on chromosomes 3B and 7B, respectively. Infection types that are different among the *Yr* genes are in bold

Wheat lines	*Yr* gene	*Pst* pathotype			
		134 E16 A + 17 + 27 +	110 E143 A +	239 E237 A- 17 + 33 +	198 E16 A + 17 + J + T +
Chromosome 3B AGG40807WHEA1/AvS.75	*YrV1*	1c	23c	3c	12c
*Yr57*/Hartog.17	*Yr57*	0	0;	3 +	;1 = cn
Rubric	*Yr4*	;1-cn	3 +	3 +	3 +
Sonora	*Yr58*	23c	3 +	3 +	3 +
Chromosome 7B AGG40807WHEA1/AvS.43	*YrV2*	2c	23c	23c	33c
*Yr67*.43	*Yr67*	11-c	12c	3c	;cn
Morocco	-	3 +	3 +	3 +	3 +
C591	*YrC591*	1c	2c	23c	1c

### Prediction of a candidate gene for *YrV1*

Five M_2_ families (M40, M71, M166, M169 and M235) of AGG40805WHEA1 segregated (ITs 0; and 2c) indicating potential knockout of *YrV1* and the presence of *YrV2* (Fig. S3). A single mutant plant from each M_2_ families retained the flanking *YrV1* marker sequences, suggesting the absence of large deletions involving the *YRV1* locus. MutRenSeq analysis comparing *NLR*-specific sequences from the wild type and five loss-of-function mutants failed to detect an *NLR* contig with sequence variation in all five mutants, indicating the possibility for *YrV1* as a non-NLR-type resistance gene. Further, the one *NLR* contig that showed SNP changes in three mutants (M166, M169, and M235), was also mapped to the long arm of CS chromosome 3B, while *YrV1* was located in the short arm.

### Comparative genomic analysis of the *YrV1* region

**Fig. 3:**
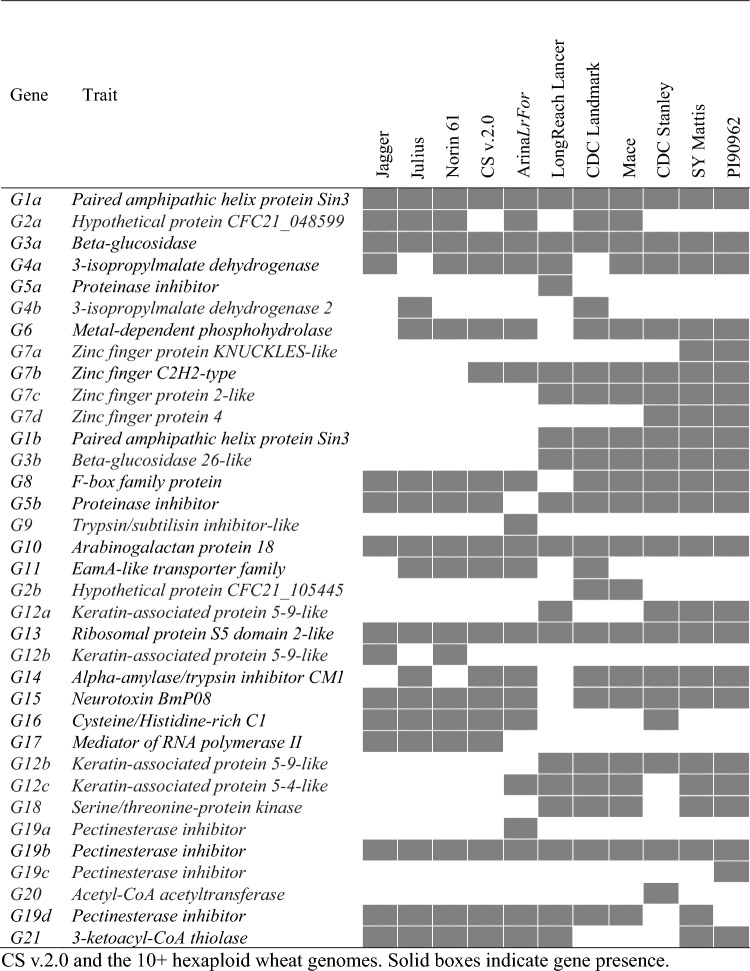
Annotated genes located in the region identified by markers flanking the *YRV1* locus in CS v.2.0 and the 10 + hexaploid wheat genomes. Solid boxes indicate gene presence

Markers *IWB72134* and *IWB71814* flanking the *YRV1* locus were positioned at 3.48 and 3.98 Mb, respectively, in CS RefSeq v.2.0. The size of the flanking region in accessions of the 10 + Wheat Genomes Project varied from 0.23 (CDC Stanley) to 0.61 (CDC Landmark) Mb; however, in CDC Stanley, Julius, Norin 61, Jagger, and Arina*LrFor* the homologous region of *YrV1* was fragmented and mapped to two different scaffolds. Within the 0.5 Mb region in CS, members of 21 different gene families were present, but none was an NLR or a kinase-encoding gene most frequently associated with rust resistance (Tong et al. 2024). Only six genes, encoding a paired amphipathic helix protein Sin3, beta-glucosidase, 3-isopropylmalate dehydrogenase 2, arabinogalactan protein 18, ribosomal protein S5 domain 2-like, and pectin esterase inhibitor, were present in CS and all 10 additional accessions. At least two copies of pectin esterase genes were present in all the lines, except CDC Stanley. Within the region of interest, all 10 + Wheat Genomes Project accessions had five genes that were missing in CS. Among them, a gene encoding a serine/threonine-protein kinase (*Stpk*) was detected in LongReach Lancer, CDC Landmark, Mace, SY Mattis, and PI 190962 (Fig. 3). However, we failed to amplify a *Stpk*-related sequence from AGG40807WHEA1 (*YrV1*) even with multiple primer sets designed from the terminal region of the CDC Landmark *Stpk* sequence. We obtained no evidence that any of the 10 + Wheat Genomes Project accessions has a resistance gene that resembles *YrV1* or *Yr57*.

### Mapping of *YrV2*

The 93 lines derived from F_3_ line AGG40807WHEA1/AvS.57 segregated 21 homozygous resistant (IT 2c), 49 segregating (IT 2c and 3 +), and 23 homozygous susceptible (with IT 3 +), indicating segregation at a single locus (χ^2^_1:2:1_- = 0.181, P_2df_ = 0.913). BSA predicted 323 SNPs in the long arm of chromosome 7B with 69 showing strong linkage with the targeted resistance. The physical interval between the SNP markers *IWB2191* and *IWB47204* was ~ 49.79 Mb in the CS reference genome v.2.0.

**Fig. 4:**
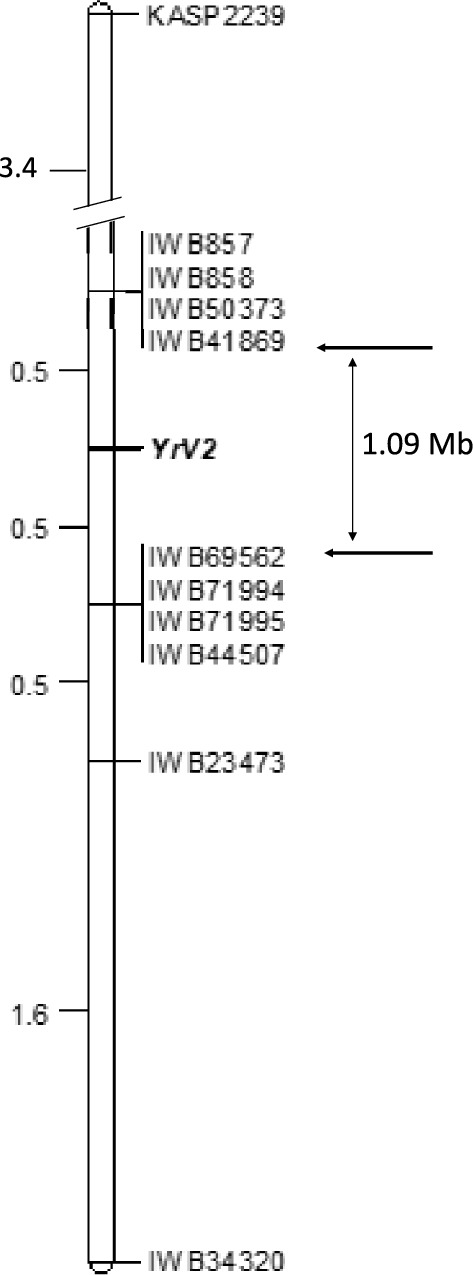
Linkage map for *YrV2* in chromosome arm 7BL on population derived from AGG40807WHEA1/AvS.57

KASP markers developed from 10 of the 69 linked SNP markers (Table S3) showed clear polymorphisms between the resistant and susceptible parents and were used to genotype the population derived from AGG40807WHEA1/AvS.57. All were within a 2.3 cM interval and *YrV2* was flanked by *IWB41869* and *IWB69562* proximally, each at a 0.5 cM distance from the *YRV2* locus (Fig. 4).

### Comparative genomic analysis of the *YRV2* region

**Fig. 5:**
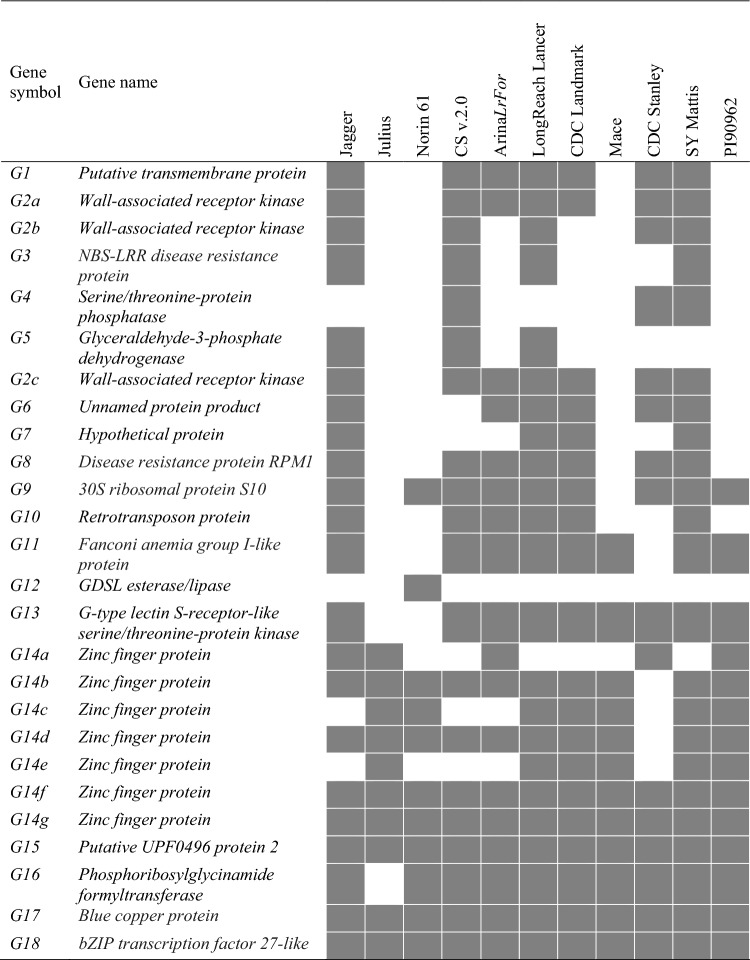
Annotated genes located in the region identified by markers flanking the *YRV2* locus in CS v.2.0 and the 10 + hexaploid wheat genomes. Solid boxes indicate gene presence

Markers *IWB41869* and *IWB69562* flanking the *YRV2* locus were positioned at 730.2 and 731.2 Mb in the CS RefSeq v.2.0. The size of the flanking region in accessions of the 10 + Wheat Genomes Project varied from 0.51 (Mace) to 1.47 (Norin 61) Mb. Within the 1 Mb region in CS, there were members of 18 different gene families, including NLR, wall-associated receptor kinase, Serine/threonine-protein phosphatase and Zinc finger protein-encoding genes known for association with disease resistance. Within the region, seven copies of a Zinc finger protein-encoding gene, and three copies of a wall-associated receptor kinase gene were present (Fig. 5).

### Relationship of *YrV2* with *Yr67* and *YrC591*

Bariana et al. (2022) mapped *Yr67* in cultivar VL Gehun 892, in the same region as *YrV2*, and it also produced a similar rippling phenotype*.* Lines AGG40807WHEA1/AvS.43 (homozygous for *YrV2*), *Yr67*.43 (*Yr67*) and C591 (*YrC591*) showed similar responses to an array of *Pst* pathotypes (IT 1-c to 23c, Table 3)*.* Despite similarities in response a conclusion on the identity of *YrV2* was not reached. However, the Indo-Gangetic origin of this gene suggests it is *Yr67*.

### Validation of KASP markers closely linked to *YrV1* and *YrV2* resistance

KASP markers *IWB71814* and *IWB69562*, closely linked to *YrV1* and *YrV2*, respectively, were used for validation (Figs. 2 and 4). Marker *IWB71814* amplified the *YrV1*-associated SNP allele in nine of 123 hexaploid wheat lines and one line in each of 15 tetraploid and 14 triticale genotypes, while *YrV2*-specific *IWB69562* allele was present in 34 hexaploid and nine tetraploid wheat accessions (Table 2). However, due to the presence of multiple resistance genes, both known and unknown, it is very hard to rule out the presence or absence of the targeted genes in these false-positive lines based on resistance responses. Further, due to recombination between these markers and the gene, the chance for false positives was high in this case. Among the 72 *T. aestivum* ssp *sphaerococcum* genotypes, only three lacked the *YrV2*-specific *IWB69562* allele (Table S4).

### Interaction of *YrV1* with *YrV2* and *Yr46*

While phenotyping F_3_ lines from the AGG40807WHEA1/AvS cross individual plants and lines with distinctive infection types 0 and ;n were recorded in materials classified as having *YrV1.* Such variation usually indicates additional modifying genes. The most likely interactions were expected to be *YrV1* + *YrV2* or *YrV1* + *Yr46*. Molecular genotyping of the 34 homozygous *YrV1YrV1* F_2:3_ lines indicated a random distribution of the *YrV2* and *Yr46* markers without an obvious association.

## Discussion

The Vavilov wheat collection is a valuable resource of genes for rust resistance. Riaz et al. (2018) and Jambuthenne et al. (2022) previously searched the collection for novel sources of APR to leaf rust and stripe rust, respectively. Here, we mapped and characterized two ASR genes (temporarily named *YrV1* and *YrV2*) effective against predominating pathotypes from the four Australian *Pst* lineages, which have distinctive avirulence/virulence patterns and belong to the global molecular groups *Pst*S0, *Pst*S1, *Pst*S10, and *Pst*S13 (Ding et al. 2021). Only a small number of formally designated ASR genes including *Yr35* and *Yr47*, are effective against all pathotypes within the four molecular groups (Bansal and Bariana, unpublished data). *YrV1* conferred a strong resistance responses (IT 0, 0; and ;n), whereas *YrV2* showed moderate response (IT 2c). The IT 0 to 0; or ;n variation among plants and lines with *YrV1* was attributed to unidentified genetic interactions, but variation in response from one test to another also suggested environmental variation.

*YRV1* was mapped to the short arm of chromosome 3B. We concluded that *YrV1* was *Yr57* based on the presence of markers *IWB72134* and *IWB71814* in sources carrying these genes. An attempt to resolve the issue by cloning *YrV1* using a MutRenSeq approach based on *NLR* gene prediction (Steuernagel et al. 2016; Marchal et al. 2018) failed to identify the gene. It is possible that *YrV1* may not be an *NLR* as it is well established that many resistance genes are not *NLRs.*

*YrV2* was mapped to chromosome arm 7BL. Genes *Yr2, Yr39* (Lin and Chen 2007)*, Yr52* (Ren et al. 2012)*, Yr59* (Zhou et al. 2014) and *Yr67/YrZh84/YrC591* (Li et al. 2006; 2009; Bariana et al. 2022) were previously located on this chromosome arm. A line carrying *Yr2* was susceptible to *Pst* pathotype 150 E16 A + and genes *Yr39, Yr52* and *Yr59* were excluded as they are high-temperature adult-plant (HTAP) resistance genes usually not detected in standard seedling tests. Although *Yr67, YrZh84* and *YrC591* were proposed to be the same gene (Bariana et al. 2022), variations was observed in the present study which might be due to differences in genetic backgrounds. However, the presence of the *YrV2* flanking markers (*IWB41869* and *IWB69562*) in *Yr67*.43 (*Yr67*) and C591 (*YrC591*) genotypes, it is highly likely that these three genes are the same. This issue will be best resolved following cloning of one or other of these genes.

Comparative genomic analysis of the *YRV1* locus using CS and the 10 + Wheat Genomes Project panel identified no *NLR* within the 0.5 Mb interval flanked by SNP markers *IWB72134* and *IWB71814* in CS, supporting the earlier suggestion that *YrV1* was not an *NLR*. The chromosome 3B region in CS also had no kinase gene, the second most common gene family causing rust resistance in cereals (Klymiuk et al. 2018). The homologous regions in the LongReach Lancer, CDC Landmark, Mace, SY Mattis, and PI 190962 genomes contained a *Stpk* gene, a gene type previously associated with wheat powdery mildew resistance (Li et al. 2024). However, our failure to amplify a *Stpk*-related sequence from accession AGG40807WHEA1, indicated the absence of *YrV1* or presence of a *Stpk* member with a highly varied sequence. In addition to *Stpk*, four other genes from the pangenome were poorly represented in the CS reference. The mosaic pattern of gene distribution and copy number variation in CS and the Wheat 10 + Genomes Project accessions indicate the possibility of a lack of synteny between AGG40807WHEA1 and sequenced hexaploid wheat genotypes for the *YRV1* locus (Fig. 3). A possible solution is to investigate the recently published database of Watkins Collection of wheat landraces (Cheng et al. 2024), where a potential candidate gene might be identified based on similarity across accessions considered to have *YrV1* based on stripe rust response data or linked markers.

A comparative genomic approach was also employed for *YrV2*, where nine candidate genes, including seven protein kinase and two *NLR* genes were predicted within the flanking marker interval in CS v.1.0 (Fig. 5). While NLR and kinase protein families were considered the major classes of disease resistance proteins for resistance to rust diseases in wheat (Tong et al. 2024), the possibility of other types cannot be dismissed. For example, the wheat leaf rust resistance gene *Lr14a* was identified as an ankyrin-transmembrane protein-encoding gene (Kolodziej et al. 2021) that would not be detected by methods used in the present work. While efforts to identify the underlying gene sequences and confirm diagnostic markers for *YrV1* and *YrV2* remain underway, we assessed the respective closely linked KASP markers based on *IWB71814* and *IWB69562* on a panel of Australian common wheat, durum wheat and triticale cultivars for application in marker-assisted selection of *YrV1* and *YrV2*. Based on the association of *YrV2*-linked marker *IWB69562* in the *T. aestivum* ssp *sphaerococcum* accessions, along with *Yr67* and *YrC591* identified in Indian and Chinese wheat accessions, the origin of *YrV2* is likely southeast Asia.

## Supplementary Information

Below is the link to the electronic supplementary material.

Supplementary file1 (DOCX 1110 KB)

Supplementary file2 (DOCX 52 KB)

## Data Availability

Not applicable.
