# Supplementary material for: Genomic analysis of two all-stage stripe rust resistance genes in the Vavilov wheat landrace AGG40807WHEA1
Source: Theor Appl Genet. 2025 Jul 9;138(8):180. doi: 10.1007/s00122-025-04965-1 (PMC12241163; doi:10.1007/s00122-025-04965-1)
Supplement: Supplementary file 1 — Supplementary file1 (DOCX 1110 KB) [file 122_2025_4965_MOESM1_ESM.docx]

AGG40691WHEA1 (WLA028)

WLA101 (AGG no unknown)

AGG40800WHEA1 (WLA146)

AGG40804WHEA1 (WLA150)

AGG40807WHEA1 (WLA153)

AGG40805WHEA1 (WLA151)

AvS


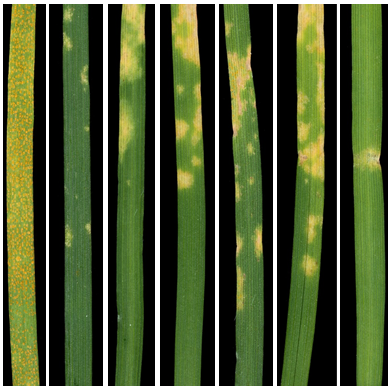


0;n

3+

;n

;n

0

;n

0;

**Fig. S1** Infection types produced by AvS and six resistant accessions from the Vavilov wheat landrace (WLA) collection when inoculated with *Pst* pathotype 150 E16 A+

198 E16 A+ 17+ J+ T+

239 E237 A- 17+ 33+

110 E143 A+

134 E16 A+ 17+ 27+

*YrV1 Yr57*

*Yr4*

*Yr58*

Morocco

*YrV1 Yr57*

*Yr4*

*Yr58*

Morocco

*YrV1 Yr57*

*Yr4*

*Yr58*

Morocco

*YrV1 Yr57*

*Yr4*

*Yr58*

Morocco


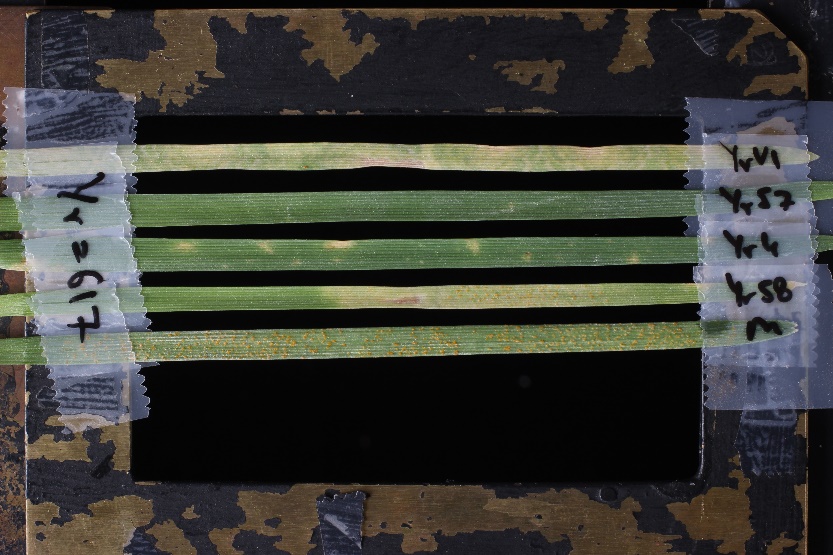

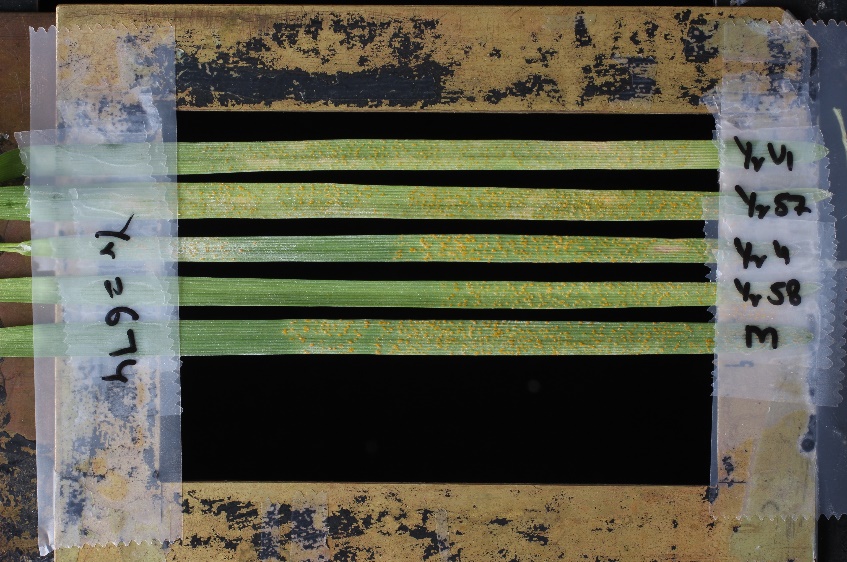

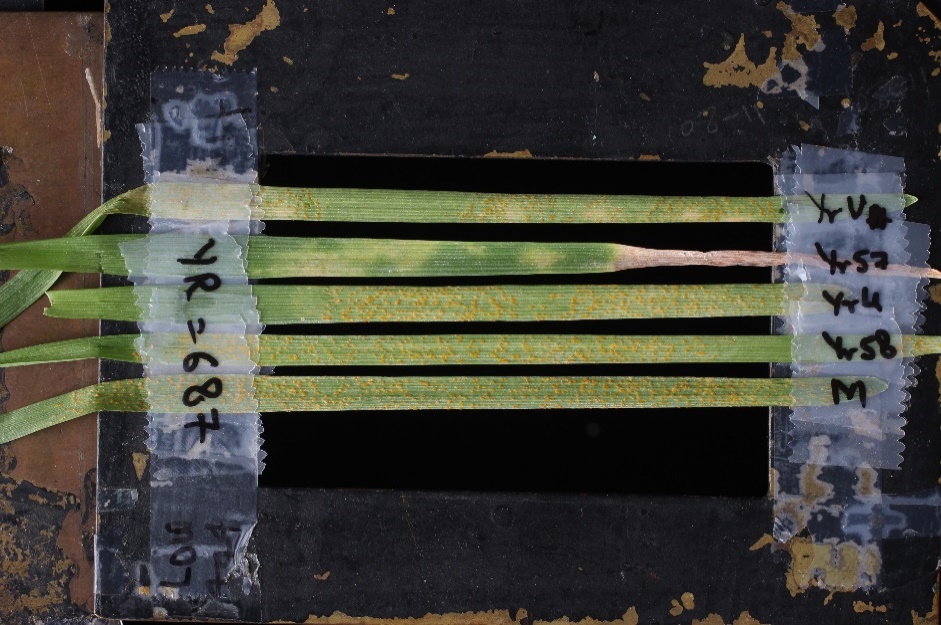


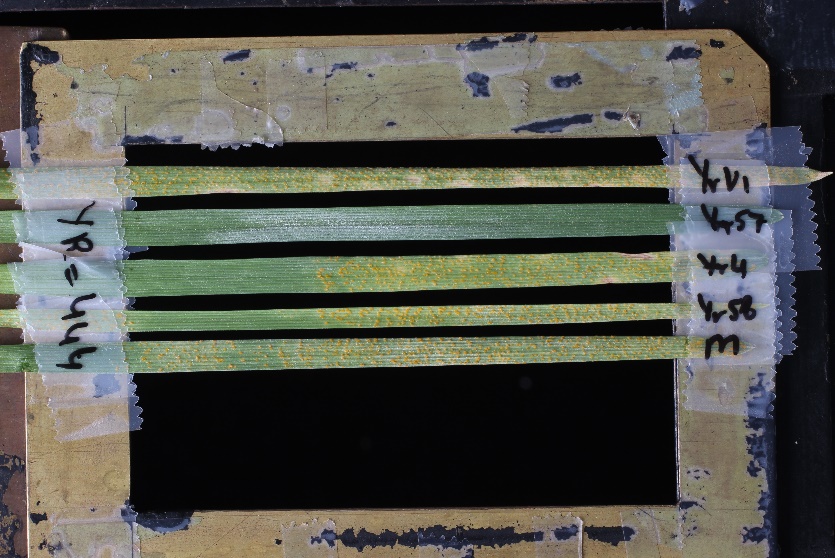


3c

3+

3+

3+

3+

+

12c

;1=cn

3+

3+

3+

+

1c

0

;1-cn 23c

3+

23c

0;

3+

3+

3+

+

**Fig. S2** Infection types produced in multi-pathotype seedling tests of lines carrying *YrV1* (AGG40807WHEA1/AvS.75), *Yr57* (*Yr57*/Hartog.17), *Yr4* (Rubric) and *Yr58* (Sonora) and Morocco (susceptible control)

WLA151 Morocco M40 M71 M166 M169 M235


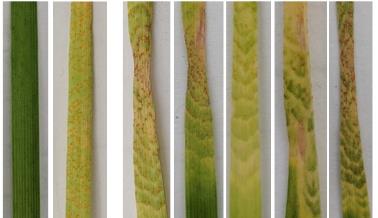


0 3+ 23c 23c 2c 2c 23c

**Fig. S3** Infection types produced by AGG40805WHEA1 (WLA151), Morocco and selected mutants when inoculated with *Pst* pathotype 150 E16 A+. All five mutants displayed the characteristic rippling response associated with *YrV2.*
