# Supplementary material for: Genomic analysis of two all-stage stripe rust resistance genes in the Vavilov wheat landrace AGG40807WHEA1
Source: Theor Appl Genet. 2025 Jul 9;138(8):180. doi: 10.1007/s00122-025-04965-1 (PMC12241163; doi:10.1007/s00122-025-04965-1)
Supplement: Supplementary file 2 — Supplementary file2 (DOCX 52 KB) [file 122_2025_4965_MOESM2_ESM.docx]

**Table S1** Primer details for markers linked with the all-stage resistance (ASR) genes *Yr1*, *Yr5*, *Yr15*, *Yr32*, and *Yr33,* and the adult-plant resistance (APR) genes *Yr18* and *Yr46*

| **Genes** | **Type** | **Marker** | **Sequence (5′– 3′)** | **Reference** |
| --- | --- | --- | --- | --- |
| *Yr1* | ASR | *stm673acag* | FP*- TAACTCACAACACGTTCTGGTCGT | Bansal et al. 2009 |
|  |  |  | RP^#^- ACACACACACACACAGAGAGAG |  |
| *Yr5* | ASR | *Yr5-insertion* | FP- CTCACGCATTTGACCATATACAACT | Marchal et al. 2018 |
|  |  |  | RP- TATTGCATAACATGGCCTCCAGT |  |
| *Yr15* | ASR | *Yr15KIN1* | FP- GGAGATAGAGCACATTACAGAC | Klymiuk et al. 2018 |
|  |  |  | RP- TTTCGCATCCCACCCTACTG |  |
| *Yr32* | ASR | *WMS198* | FP- CACGCTGCCATCACTTTTAC | Eriksen et al. 2004 |
|  |  |  | RP- TTGAAGTGGTCATTGTTGCT |  |
| *Yr33* | ASR | *GWM437* | FP- GATCAAGACTTTTGTATCTCTC | Zhou et al. 2022 |
|  |  |  | RP- GATGTCCAACAGTTAGCTTA |  |
| *Yr18* | APR | *Lr34-GS* | FP- GGGAGCATTATTTTTTTCCATCATG | Krattinger et al. 2011 |
|  |  |  | RP- ACTTTCCTGAAAATAATACAAGCA |  |
| *Yr46* | APR | *SNP1-TM4* | FP- TCATCATCGGCAGGATCCTGCTTC | E. Lagudah, unpublished (Pers. ) |
|  |  |  | RP- TCATCATCGGCAGGATCCTGCTTG |  |

*FP - forward primer; ^#^RP - reverse primer

**Table S2** KASP markers used for mapping *YrV1* on chromosome arm 3BS.

| **Primer name** | **Sequence** |
| --- | --- |
| *IWB72133* | FAM-TCCTTCAACGTCGTGGGT |
|  | HEX-TCCTTCAACGTCGTGGGC |
|  | COM-AGAAAGTGAGGGTGAGGCG |
| *IWB11112* | FAM-TCCTTCAACGTCGTGGGT |
|  | HEX-TCCTTCAACGTCGTGGGC |
|  | COM-AGAAAGTGAGGGTGAGGCG |
| *IWB72134* | FAM-TGATGAGGAAAGCACTCAAAGA |
|  | HEX-TGATGAGGAAAGCACTCAAAGG |
|  | COM-CACCGGATTCACTTCCTGAC |
| *IWB71814* | FAM-GGTAGATCACATGGATGGCTTT |
|  | HEX-GGTAGATCACATGGATGGCTTC |
|  | COM-GCGCTTGGTTATAATCCCATG |
| *IWB64176* | FAM-GCTCATTATTTTCAGGCAGCGA |
|  | HEX-GCTCATTATTTTCAGGCAGCGC |
|  | COM-GCCTGCTGACCCTAAAACAAG |
| *IWB71498* | FAM-AGGTTAGGGGAAGAAGCAGA |
|  | HEX-AGGTTAGGGGAAGAAGCAGG |
|  | COM-CGACCCGATGTACGACATGA |
| *IWB10800* | FAM-CCGTAAGCTATTTTGTGTTCAGATA |
|  | HEX-CCGTAAGCTATTTTGTGTTCAGATG |
|  | COM-GGCCTACCAACAACCAACCA |
| *IWB8467* | FAM-ACACAATGACAACACTGGGT |
|  | HEX-ACACAATGACAACACTGGGC |
|  | COM-GAGGACGGTGATGCTTGTGA |

**Table S3** SNP and derived KASP markers used for mapping *YrV2* on chromosome arm 7BL.

| **Primer name** | **Sequence** |
| --- | --- |
| *IWB857* | FAM-GCTTCGGTTCCTCTCGTCT |
|  | HEX-GCTTCGGTTCCTCTCGTCC |
|  | COM-GGGGCATATGAATATACCTGAACG |
| *IWB858* | FAM-AATCATGCGCTCTTTAGGGAT |
|  | HEX-AATCATGCGCTCTTTAGGGAC |
|  | COM-ATGCTGACCAGAATTTTGTCTTT |
| *IWB50373* | FAM-ACTTTGATGTTATCCGCAATCCA |
|  | HEX-ACTTTGATGTTATCCGCAATCCG |
|  | COM-TTTCTTCTTGACAGTGCCCAG |
| *IWB41869* | FAM-CAGTGTTTCTTTGCATGGTTCA |
|  | HEX-CAGTGTTTCTTTGCATGGTTCA |
|  | COM-CAGTGTTTCTTTGCATGGTTCG |
| *IWB69562* | FAM-GCGAGATCCTCATGGCTCT |
|  | HEX-GCGAGATCCTCATGGCTCG |
|  | COM-AGGCTGAAGTTTCTCGAAGG |
| *IWB71994* | FAM-GGCGGCGATGATGATGACA |
|  | HEX-GGCGGCGATGATGATGACG |
|  | COM-CCACCCTAGAGAAGCGGAAA |
| *IWB71995* | FAM-GGTGGCGCTCTGATCCGT |
|  | HEX-GGTGGCGCTCTGATCCGC |
|  | COM-GCGCCCATCGACATTAAACA |
| *IWB44507* | FAM-TGAAGAAGCTGGAGTCGAGT |
|  | HEX-TGAAGAAGCTGGAGTCGAGC |
|  | COM-TAGTCCTTGCCAAAGTCCGC |
| *IWB23473* | FAM-CAATCTTCTTGCTGAACCCAAAT |
|  | HEX-CAATCTTCTTGCTGAACCCAAAC |
|  | COM-GGCAAGACAATGTGACAACC |
| *IWB34320* | FAM-TTGCCAGATTGCCCAAGCA |
|  | HEX-TTGCCAGATTGCCCAAGCG |
|  | COM-CACAGCCCGACCATCTCA |

**Table S4** *Triticum sphaerococcum* germplasm used for testing KASP marker *IWB69562* linked with *YrV2*

| ***T. sphaerococcum* (AGG No.)** | ***IWB69562* Allele** |
| --- | --- |
| AGG866WHEA1 | T |
| AGG879WHEA1 | T |
| AGG967WHEA1 | T |
| AGG1201WHEA1 | - |
| AGG1779WHEA1 | T |
| AGG1906WHEA1 | T |
| AGG2311WHEA1 | T |
| AGG3693WHEA1 | T |
| AGG3860WHEA1 | T |
| AGG3861WHEA1 | T |
| AGG3862WHEA1 | T |
| AGG3863WHEA1 | T |
| AGG3864WHEA1 | T |
| AGG3865WHEA1 | T |
| AGG3866WHEA1 | T |
| AGG3867WHEA1 | T |
| AGG3868WHEA1 | T |
| AGG3869WHEA1 | T |
| AGG3870WHEA1 | T |
| AGG3871WHEA1 | T |
| AGG3873WHEA1 | T |
| AGG3875WHEA1 | T |
| AGG3876WHEA1 | T |
| AGG3877WHEA1 | T |
| AGG4637WHEA1 | T |
| AGG4641WHEA1 | T |
| AGG4642WHEA1 | T |
| AGG4649WHEA1 | T |
| AGG4650WHEA1 | T |
| AGG4671WHEA1 | T |
| AGG4718WHEA1 | T |
| AGG4719WHEA1 | T |
| AGG4866WHEA1 | T |
| AGG4867WHEA1 | T |
| AGG7977WHEA1 | T |
| AGG11453WHEA1 | T |
| AGG15265WHEA1 | T |
| AGG15952WHEA1 | T |
| AGG16309WHEA1 | T |
| AGG16454WHEA1 | G |
| AGG18373WHEA1 | T |
| AGG18374WHEA1 | T |
| AGG18375WHEA1 | T |
| AGG18376WHEA1 | T |
| AGG19387WHEA1 | T |
| AGG19388WHEA1 | T |
| AGG19389WHEA1 | T |
| AGG19390WHEA1 | T |
| AGG19391WHEA1 | T |
| AGG20265WHEA1 | T |
| AGG21034WHEA1 | T |
| AGG21757WHEA1 | T |
| AGG21825WHEA1 | G |
| AGG22404WHEA1 | T |
| AGG22405WHEA1 | T |
| AGG22406WHEA1 | T |
| AGG22407WHEA1 | T |
| AGG28812WHEA1 | T |
| AGG28813WHEA1 | T |
| AGG28814WHEA1 | T |
| AGG28815WHEA1 | T |
| AGG28816WHEA1 | T |
| AGG28817WHEA1 | T |
| AGG28818WHEA1 | T |
| AGG28819WHEA1 | G |
| AGG28820WHEA1 | T |
| AGG28821WHEA1 | T |
| AGG28822WHEA1 | T |
| AGG28823WHEA1 | T |
| AGG33889WHEA1 | T |
| AGG33890WHEA1 | T |
| AGG36731WHEA1 | T |
| AGG40807WHEA1 (Positive control for *YrV2*) | T |
| Avocet S (Negative control) | G |
| - missing data |  |

**Table S5** Seedling stripe rust responses of 38 accessions from the Vavilov collection to single pathotypes from pre- and post-2002 *Pst* lineages. Six accessions resistant to both pathotypes are highlighted in yellow, while the post-2002 alone is in green. “^§”^data from a previous study (L. Hickey, unpublished) and * represents data from the current study.

| **S. No.** | **AGG No.** | **UQ number** | **Post-2002**  **(134 E16 A+ 17+ 27+)^§^** | **Pre-2002 (104 E137 A+ )*** |
| --- | --- | --- | --- | --- |
| 1 | AGG40664WHEA1 | WLA-001 | 1=c | 3+ |
| 2 | AGG40665WHEA1 | WLA-002 | 0 | 3+ |
| 3 | AGG40686WHEA1 | WLA-023 | ;n | 3 |
| 4 | AGG40691WHEA1 | WLA-028 | ;n1= | ;1 |
| 5 | AGG40718WHEA1 | WLA-055 | ;n1 | 3 |
| 6 | AGG40720WHEA1 | WLA-057 | ;n | 2+ |
| 7 | AGG40721WHEA1 | WLA-058 | 1n; | 3+ |
| 8 | AGG40730WHEA1 | WLA-067 | 0; | 3+ |
| 9 | AGG40731WHEA1 | WLA-068 | 1c | 3+ |
| 10 | AGG40736WHEA1 | WLA-073 | ;n1= | 3 |
| 11 | AGG40743WHEA1 | WLA-080 | 0; | 3+ |
| 12 | Not available | WLA-101 | ;n1= | ;cn |
| 13 | Not available | WLA-102 | ;n1= | 3 |
| 14 | AGG40772WHEA1 | WLA-115 | 1=c | 2+ |
| 15 | AGG40774WHEA1 | WLA-117 | ;n1= | 3 |
| 16 | AGG40800WHEA1 | WLA-146 | 0; | 1- |
| 17 | AGG40801WHEA1 | WLA-147 | ;n | 3 |
| 18 | AGG40803WHEA1 | WLA-149 | 0; | 3 |
| 19 | AGG40804WHEA1 | WLA-150 | ;n | 1+ |
| 20 | AGG40805WHEA1 | WLA-151 | 0; | ;1 |
| 21 | AGG40806WHEA1 | WLA-152 | 0; | 3+ |
| 22 | AGG40807WHEA1 | WLA-153 | 0; | ;1 |
| 23 | AGG40808WHEA1 | WLA-154 | ;n | 3+ |
| 24 | AGG40861WHEA1 | WLA-211 | ;n1= | 3+ |
| 25 | AGG40862WHEA1 | WLA-212 | ;n | 3 |
| 26 | AGG40865WHEA1 | WLA-215 | 0; | 3 |
| 27 | AGG40887WHEA1 | WLA-238 | 1nc | 3 |
| 28 | AGG40896WHEA1 | WLA-250 | ;n | 3 |
| 29 | AGG40916WHEA1 | WLA-271 | 1=n | 3+ |
| 30 | AGG40929WHEA1 | WLA-286 | 1cn | 3 |
| 31 | AGG40930WHEA1 | WLA-287 | 1cn | 3+ |
| 32 | AGG40936WHEA1 | WLA-293 | ;nc | 3+ |
| 33 | AGG40939WHEA1 | WLA-296 | 1-c | 3+ |
| 34 | AGG40949WHEA1 | WLA-306 | 0; | 3 |
| 35 | AGG40951WHEA1 | WLA-308 | 0; | 3+ |
| 36 | AGG40952WHEA1 | WLA-309 | ;n1= | 3+ |
| 37 | AGG40956WHEA1 | WLA-313 | ;n | 3+ |
| 38 | AGG40957WHEA1 | WLA-314 | ;cn | 3+ |
|  |  | Morocco (control) | 3+ | 3+ |

**Table S6** Genotypes of 147 F_3_ lines from the cross AGG40807WHEA1/AvS for reaction to *Pst* pathotype 150 E16 A+.

| **Genotype** | **Expected frequency** | **Observed** | **Expected** | ***χ^2^ value** |
| --- | --- | --- | --- | --- |
| ^#^AA-- | 1/2/1 | 34 | 36.8 | 0.210 |
| AaBB^Ψ^ | 2 | 15 | 18.4 | 0.619 |
| AaBb^Ψ^ | 4 | 44 | 36.8 | 1.430 |
| Aabb | 2 | 17 | 18.4 | 0.102 |
| aaBB | 1 | 7 | 9.2 | 0.520 |
| aaBb | 2 | 21 | 18.4 | 0.375 |
| aabb | 1 | 9 | 9.2 | 0.003 |
| **χ**^2^_4:2:4:2:1:2:1_ | | | | 3.265 |

***χ**^2^ = 3.265, df = 6, P = 0.775. ^#^ Segregation pattern of the B component unknown due to masking of its intermediate effect by A with strong resistance effect. ^Ψ^AaBB and AaBb were distinguished based on the detection of susceptible plants in the latter group.
